# A Potential Novel Spontaneous Preterm Birth Gene, *AR*, Identified by Linkage and Association Analysis of X Chromosomal Markers

**DOI:** 10.1371/journal.pone.0051378

**Published:** 2012-12-05

**Authors:** Minna K. Karjalainen, Johanna M. Huusko, Johanna Ulvila, Jenni Sotkasiira, Aino Luukkonen, Kari Teramo, Jevon Plunkett, Verneri Anttila, Aarno Palotie, Ritva Haataja, Louis J. Muglia, Mikko Hallman

**Affiliations:** 1 Department of Pediatrics, Institute of Clinical Medicine, University of Oulu, Oulu, Finland; 2 Department of Obstetrics and Gynecology, Helsinki University Central Hospital, Helsinki, Finland; 3 Stanford University School of Medicine, Palo Alto, California, United States of America; 4 Institute for Molecular Medicine Finland (FIMM), University of Helsinki, Helsinki, Finland; 5 Wellcome Trust Sanger Institute, Cambridge, United Kingdom; 6 Department of Medical Genetics, Haartman Institute, University of Helsinki and Helsinki University Central Hospital, Helsinki, Finland; 7 The Broad Institute of MIT and Harvard, Cambridge, Massachusetts, United States of America; 8 Biocenter Oulu, University of Oulu, Oulu, Finland; 9 Center for Prevention of Preterm Birth, Perinatal Institute, Cincinnati Childreńs Hospital Medical Center, Cincinnati, Ohio, United States of America; Sanjay Gandhi Medical Institute, India

## Abstract

Preterm birth is the major cause of neonatal mortality and morbidity. In many cases, it has severe life-long consequences for the health and neurological development of the newborn child. More than 50% of all preterm births are spontaneous, and currently there is no effective prevention. Several studies suggest that genetic factors play a role in spontaneous preterm birth (SPTB). However, its genetic background is insufficiently characterized. The aim of the present study was to perform a linkage analysis of X chromosomal markers in SPTB in large northern Finnish families with recurrent SPTBs. We found a significant linkage signal (HLOD  = 3.72) on chromosome locus Xq13.1 when the studied phenotype was being born preterm. There were no significant linkage signals when the studied phenotype was giving preterm deliveries. Two functional candidate genes, those encoding the androgen receptor (AR) and the interleukin-2 receptor gamma subunit (IL2RG), located near this locus were analyzed as candidates for SPTB in subsequent case-control association analyses. Nine single-nucleotide polymorphisms (SNPs) within these genes and an *AR* exon-1 CAG repeat, which was previously demonstrated to be functionally significant, were analyzed in mothers with preterm delivery (*n* = 272) and their offspring (*n* = 269), and in mothers with exclusively term deliveries (*n* = 201) and their offspring (*n* = 199), all originating from northern Finland. A replication study population consisting of individuals born preterm (*n* = 111) and term (*n* = 197) from southern Finland was also analyzed. Long *AR* CAG repeats (≥26) were overrepresented and short repeats (≤19) underrepresented in individuals born preterm compared to those born at term. Thus, our linkage and association results emphasize the role of the fetal genome in genetic predisposition to SPTB and implicate *AR* as a potential novel fetal susceptibility gene for SPTB.

## Introduction

Preterm birth (birth before 37 completed weeks of gestation) is a major global healthcare problem, affecting approximately 13 million people every year [1]. Approximately 12% of all infants are born preterm in the United States; this figure is 6% in Europe [2]. Seventy percent of mortality and 75% morbidity in the neonatal period are caused by preterm birth, and preterm infants also face a substantial risk of several serious morbidities that may have life-long consequences [3]. Multiple gestation, maternal chronic diseases, fetal malformations, and infections are among the factors known to increase the risk of preterm birth [4]. Preterm birth tends to recur in families, with mothers with previous preterm deliveries being at a significantly increased risk of preterm delivery in subsequent pregnancies [5]–[9]. Preterm birth also occurs across generations and sibships, with the risk of preterm delivery shared between mothers and daughters, and sisters [7], [8], [10]–[13]. Aggregation of preterm deliveries in families suggests a significant role for genetic factors in preterm birth. Approximately 70% of all preterm births are spontaneous, and no environmental risk factor can be identified for most of these births [4]. Thus, it is likely that several spontaneous preterm births (SPTBs) are explained by genetic factors.

Because initiation of parturition is preceded by physiological changes occurring in both the mother and fetus, it is possible that both maternal and fetal genes may predispose to preterm birth [14]. Most large population-based studies suggest that maternal genetic factors play the most significant role with little or a substantially smaller contribution of the fetal genes [8], [12], [13]. Some genetic modeling studies have further estimated that, in addition to environmental factors, the variation in gestational age is explained by both maternal and fetal genetic factors [15]–[17], while other studies have failed to find a significant contribution of the fetal genes [12], [13]. Overall, these studies suggest that the genetic influences of SPTB are most likely to be either direct effects of maternal genes acting in the mother, of maternally-inherited mitochrondrial genes acting in the fetus, or of maternally-inherited fetal genes. Knowledge about the actual genes predisposing to SPTB is insufficient. So far, the significance of specific candidate genes in SPTB has been assessed in numerous case-control studies [18], focusing mostly on genes involved in the infection and inflammation pathway, such as those encoding tumor necrosis factor α (TNF-α) and several interleukins [19]–[23]. Although significant associations of both maternal and fetal genetic factors have been reported in several studies, most of the studies have been limited in several respects, including small sample sizes, inconsistent phenotype definitions, and failure to replicate in subsequent studies and across populations [18]. Recent more comprehensive association studies have implied a role for genes involved in multiple pathways in SPTB emphasizing the heterogeneity in the SPTB phenotype; these associating genes include the maternal *FSHR* and *TIMP2* genes, encoding follicle-stimulating hormone receptor and tissue inhibitor of metalloproteinase 2, respectively [24], [25], and the fetal *IL6R* and *COL5A2* genes, encoding interleukin-6 receptor and collagen type V, alpha 2, respectively [25], [26]. To date, genome-wide case-control association analyses of SPTB have not been reported.

Recently, we reported the results of the first linkage analysis of SPTB in seven large northern Finnish families with recurrent SPTBs using a high-density array of autosomal markers [27]. The gene encoding insulin-like growth factor receptor 1 (IGF-1R) was identified as a potential SPTB-predisposing gene. In the present study, linkage analysis was performed for these same families using X chromosomal markers. After linkage analysis, two genes, encoding the androgen receptor (AR) and the interleukin-2 receptor gamma subunit (IL2RG), located near a significant linkage signal were further analyzed as candidates for SPTB in case-control populations originating from northern and southern Finland.

## Materials and Methods

### Ethics Statement

The study was approved by the Ethics Committees of Oulu University Hospital and Helsinki University Central Hospital. Written informed consent was obtained from the subjects participating in the study.

### Study Populations

#### Definition of SPTB and inclusion criteria for the SPTB cases

SPTB was defined as spontaneous preterm birth occurring before 36 completed weeks of gestation. Preterm deliveries occurring both with intact membranes and those following preterm premature rupture of fetal membranes (PPROM, defined as leakage of amniotic fluid as the presenting symptom before the onset of contractions) were included among the SPTBs. Deliveries involving known risk factors for preterm birth (multiple gestation, polyhydramnios, septic or acute systemic infection of the mother, diseases of the mother that possibly affect the pregnancy outcome including those influencing cardio-pulmonary, hepato-renal, or endocrine functions, and chronic inflammatory disease, alcohol or narcotic use, accidents, and fetuses with congenital anomalies) and all medically indicated preterm births without labor (preeclampsia, intrauterine growth restriction, and placental abruption) were excluded. All analyzed subjects were of Finnish origin. The overall rate of preterm birth is approximately 5.5% in Finland [28]. Considering that approximately 70% of all preterm deliveries are spontaneous and that some spontaneous deliveries were further excluded from our study due to border-line gestational ages (birth at gestational age ranging from 36 weeks + 0 days to 36 weeks + 6 days; approximately 35% of all preterm births), multiple gestation (approximately 30% of all preterm births and 15% of all deliveries with a spontaneous onset) or any of the other above-mentioned exclusion criteria (leading to exclusion of approximately 15% of all deliveries), the proportion of all preterm deliveries fulfilling our selection criteria is estimated to be approximately 20%, which corresponds to approximately 1.1% of all births in Finland.

#### Families in Linkage Analysis

**Figure 1:**
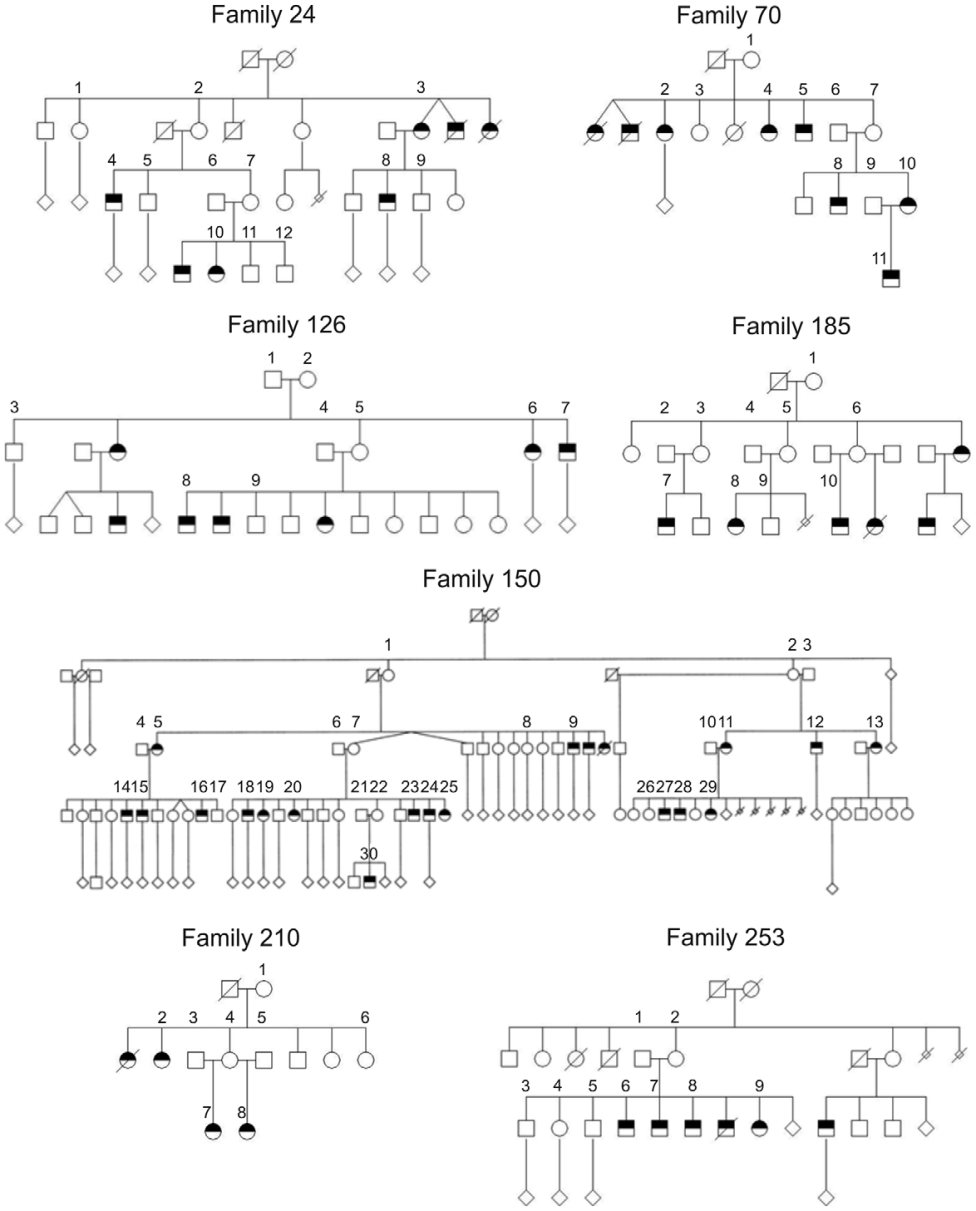
Pedigrees of the seven families with recurrent spontaneous preterm births analyzed in the linkage analysis of X chromosomal markers. Individuals born preterm are shown by half-black symbols. Squares represent males and circles females. Individuals analyzed in the linkage analysis are indicated by a number (*n* = 89). The diamonds denote an unspecified number of term infants, the smaller diamonds with a line through the symbol represent a miscarriage, and the lines through the other symbols indicate that the person is deceased. Gestational ages and years of birth for the family members born preterm are shown in Table S1. Linkage analysis was performed in two settings: 1) being spontaneously born preterm as the phenotype (affected offspring phenotype, *n_affected_*  = 41), and 2) giving spontaneous preterm births as the phenotype (affected mother phenotype, *n_affected_*  = 21). These pedigrees have been previously shown in the supplementary material of our linkage study of SPTB performed using autosomal markers [27].

The families used in the current study were the same as those used in our recently published linkage study performed with autosomal markers [27]. Families with recurrent SPTB were selected retrospectively from birth diaries of Oulu University Hospital during 1973−2003, and prospectively during 2003−2005. Family interviews revealed 20 large families with SPTBs in two or more generations or in first cousins. To ensure that the families were nonconsanguineous, a genealogical study was performed according to published criteria [29]. An examination of the microfiche copies available in the provincial and national archive of Finland did not reveal close consanguinity or common residence dating from the 17^th^ century among the families. Finally, the families with apparent maternal inheritance of the SPTB phenotype were selected for the study because most previous family studies suggest that maternal genes or maternal genes acting in the fetus (rather than paternal) influence the duration of pregnancy and the inheritance of preterm delivery [8], [12], [13], [16]. Whole-blood DNA samples (*n* = 89) were collected from as many family members as possible. The pedigrees of the seven families in the linkage analysis are depicted in Figure 1; gestational ages and years of birth for the family members born preterm are shown in Table S1.

#### Study Sample in the Initial Case-Control Association Analysis

Mothers with one or more spontaneous preterm deliveries and their preterm offspring (born at <36 gestational weeks) born in the Oulu University Hospital region (northern Finland) were selected for the study during the same time period as the linkage analysis families. Cases included mothers and their offspring from both families with multiple (two or more) SPTBs as well as those with a single SPTB in the family. For 39 families with multiple SPTBs, DNA samples of two or more individuals born preterm were available. To include deliveries involving as few as possible other than genetic risk factors for SPTB, a single individual born preterm was selected for analysis from these families according to the following low-risk criteria: 1) age of the mother between 20 and 35 years at the time of the birth of the individual born preterm (criterion applied because low and high maternal ages are risk factors for preterm birth [4]; 9 individuals selected using this criterion), and 2) no deliveries within the preceding 2 years, or the longest time interval from the previous delivery (criterion applied because short inter-pregnancy interval is a risk factor for preterm birth [4]; 22 individuals selected using this criterion). If these criteria were not applicable, a female individual was selected over a male individual (criterion applied because a somewhat higher proportion of male fetuses are born preterm compared to female fetuses [30]; 8 individuals selected using this criterion). None of the SPTB cases were members of any of the linkage analysis families. The clinical characteristics of the case study population are shown in Table 1. Whole-blood or buccal samples were obtained from cases (141 whole-blood and 131 buccal samples of SPTB mothers; 104 whole-blood and 165 buccal samples of SPTB offspring), and when available, also from fathers (*n* = 226; 117 whole-blood and 109 buccal samples). The control population comprised mothers with exclusively at least three term (gestational age ≥37 wk) deliveries (*n* = 201) without any pregnancy- or labor-associated complications (intrauterine growth restriction, placental abruption, polyhydramnios, preeclampsia, fetuses with congenital anomalies, requirement of special care of the newborn) and their singleton offspring (*n* = 199; 110 males and 89 females) collected prospectively following delivery in the Oulu University Hospital during 2004−2007. The selection criterion “exclusively at least three term deliveries” was applied because women who have had several uncomplicated term deliveries without any preterm deliveries are likely to be at low risk to give birth preterm in subsequent pregnancies and thus most unlikely to carry genetic factors that predispose to SPTB. Whole-blood or buccal samples were obtained from controls (143 whole-blood and 58 buccal samples of control mothers; 199 whole-blood samples of control offspring).

**Table 1:** Clinical characteristics of the case study populations of spontaneous preterm birth.

	Initial population from northern Finland		Replication population from southern Finland
Characteristic	Offspring	Mothers	Offspring
Total *n*	269^a^	272^a^	111
Single spontaneous preterm delivery in the family, *n*	194	199	95
Two spontaneous preterm deliveries in the family, *n*	60	57	16
3–5 spontaneous preterm deliveries in the family, *n*	15	16	0
Gestational age, wk	31.8±2.99 (24.1–35.9)^b^		34.0±1.92 (27.6–35.9)^b^
Gestational age <32 wk, *n*	109		15
Gestational age ≥32 wk, *n*	160		96
Birth weight, g	1,864±618 (538–3,070)^b^		2,361±492 (1,130–3,598)^b^
PPROM, yes/no	111/158		67/44
Male/female	149/120		56/55
Maternal smoking during pregnancy, yes/no/unknown	34/170/65	33/175/64	6/105/0

a Mismatch in the numbers of mothers and offspring is due to occasional low quality of DNA samples.

b Mean ± standard deviation (range).

#### Replication Study Sample for the Case-Control Association Analysis

Due to a significant linkage signal with the affected offspring phenotype and association of a fetal polymorphism in the initial case-control population, a replication study sample consisting of individuals born preterm (at <36 gestational weeks; *n* = 111; 56 males and 55 females) and term (*n* = 197; 94 males and 103 females) collected in the Helsinki University Central Hospital (southern Finland) during 2005−2008 was analyzed. The cases (SPTB offspring) were selected using the same criteria as described for the initial study sample. Similarly to the initial offspring control population, the replication control population was comprised of individuals born at gestational age ≥37 wk for mothers who had experienced no pregnancy-or labor-associated complications. However, it should be noted that the replication control study sample did not meet one of the criteria for the selection of the initial controls, that is, the occurrence of exclusively at least three term deliveries in the family. Whole-blood samples were obtained from cases and controls.

### DNA Sample Preparation

Genomic DNA was extracted from whole blood or buccal cells using standard methods as described [27]. Buccal DNA was whole-genome amplified (WGA) using the Illustra GenomiPhi V2 DNA Amplification kit (GE Healthcare Sciences, Cardiff, UK) and purified using Illustra Microspin G-50 columns (GE Healthcare Sciences). Nontemplate reactions were included in the WGA reactions to control for contamination. The quality of the WGA samples was controlled in the set-up stage by genotyping amplified DNA samples in duplicate reactions simultaneously with the corresponding unamplified samples and whole-blood DNA samples obtained from the same individuals using a test set of single-nucleotide polymorphisms (SNPs); this yielded >99% consistent genotypes. Samples with low genotyping success rate were further excluded from analyses.

### Genotyping and Data Analysis in the Linkage Study

DNA samples for the linkage analysis were genotyped in the Broad Institute Center for Genotyping and Analysis (CGA) using the Affymetrix Genome-Wide Human SNP Array 5.0Kb.

Before linkage analysis, the Affymetrix SNP array data was processed using PLINK, v. 1.02 [31]. A 1-Mb-to-1 cM map was used as justified before [32]. SNPs occurring at a minor allele frequency (MAF) <0.08 in the studied subjects, genotyping failure >0.1, Mendelian errors, or deviation from HWE (*p*<0.001) were excluded. The absence of Mendelian errors was confirmed using Pedcheck [33]. After these pruning steps, 4,870 X chromosomal SNPs with an average spacing of 0.031 Mb remained for linkage analysis.

Linkage analysis of X chromosomal markers was performed in two settings: 1) being spontaneously born preterm as the phenotype (affected offspring phenotype, *n_affected_*  = 41), and 2) giving spontaneous preterm births as the phenotype (affected mother phenotype, *n_affected_*  = 21). Two-point linkage analysis was applied to avoid bias potentially arising in multipoint analyses due to markers displaying linkage disequilibrium (LD) or missing parental genotype data [34]. We used a dominant, low-penetrance model with a disease allele frequency set to 0.001 and penetrances of 0.001 for the homozygotes, 0.001 for the heterozygotes and 0 for the wild-type homozygotes. The effect of misspecifying the actual mode of inheritance (MOI) is minimized in this model. Therefore, this model is nearly equivalent to a model-free analysis while the high power of parametric analysis to detect linkage is retained [35], [36]. Analyze, v. 1.9.3. BETA [37], which uses FASTLINK 4.1P for calculation of pedigree likelihoods, was used in parametric two-point linkage analysis of SPTB. Heterogeneity LOD (HLOD) scores and the estimates of proportion of linked families (α) and recombination fraction (θ) were used to assess parametric linkage in the presence of heterogeneity. An HLOD score >3.3 was considered as a significant signal of linkage.

### Selection of Genes For the Case-Control Study

Due to a significant linkage signal for marker rs6525299 on chromosome locus Xq13.1, we screened the region (∼5 Mb) surrounding the linkage peak for the presence of plausible candidate genes for SPTB and chose to analyze two genes near this locus (*AR* and *IL2RG*, located upstream and downstream from rs6525299, respectively, each at a distance of ∼2 Mb) as candidates for SPTB. The *AR* and *IL2RG* genes were chosen for analysis based on their function: *IL2RG*, encoding for a signaling component (the common gamma chain) of several interleukin receptors with essential roles in the immunity [38], emerged as an interesting candidate because infections are estimated to be involved in approximately 30–40% of preterm births [39]; *AR* emerged as an interesting candidate due to the role of androgen receptor in the development of several tissues and due to involvement of androgens in the biosynthesis of estrogen, which is essentially involved in the regulation of parturition initiation [40], [41]. Altogether 65 and 41 SNPs (at an average SNP spacing of 0.03 Mb) analyzed in the Affymetrix linkage array were located within the interval spanning from the most significant linkage peak (rs6525299) to the *AR* and *IL2RG* genes, respectively. In linkage analysis, suggestive or nearly suggestive linkage signals (HLODs 1.5−2.7) were detected for several SNPs located between the linkage peak and these two genes, while some of the SNPs located in this region showed no signals (data not shown). According to 1000 Genomes Project sequence data, SNP rs6525299 (the marker yielding the significant linkage signal) occurs at a MAF of 0.16 in the Finnish population, while many of the SNPs of the Affymetrix linkage array located between this SNP and the *AR* and *IL2RG* genes occur at a MAF of 0.25−0.35. Ability to detect LD between a SNP and a given genetic variant is influenced by allele frequencies of these variants [42]–[44]. Thus, a SNP may display linkage with a given genetic variant even though other markers located close to this variant show weaker linkage signals.

### Selection of Polymorphisms and Genotyping in the Case-Control Study

Tagging SNPs (tSNPs) were selected for the *AR* and *IL2RG* genes using HapMap data (release23a/phaseII) from the CEU (CEPH; Utah residents with ancestry from northern and western Europe) population. Using pairwise tagging with MAF and *r^2^* cutoffs of 0.05 and 1.0, respectively, six tSNPs (rs5918757, rs5919393, rs2361634, rs5918762, rs12014709, and rs5031002) were identified for the *AR* gene. In addition, two exonic *AR* polymorphisms were further selected for analysis due to previously reported associations of these polymorphisms to pregnancy-related conditions and/or due to reported functional consequences: the *AR* exon-1 SNP rs6152 (Glu213Glu) was selected because it was previously shown to associate with recurrent spontaneous abortions [45], and the exon-1 CAG repeat polymorphism (CAG*_n_*) was selected because previous studies have shown that this polymorphism affects the AR-mediated transactivation [46], correlates with the concentration of androgens [47], and associates with recurrent spontaneous abortions [48]. For the *IL2RG* gene, no tSNPs were identified even with a MAF cutoff of 0. Therefore, we analyzed all common SNPs (MAF >0.05) indicated for this gene (rs4612655, rs4073907, rs11574625, rs7880291, and rs28743771) in the dbSNP database (www.ncbi.nlm.nih.gov/SNP). After validation of the genotyping procedure, one SNP in *AR* (rs2361634) and two SNPS in *IL2RG* (rs4073970 and rs11574625) were excluded from the analysis.

SNP genotyping was performed using the SNaPshot Multiplex kit (Applied Biosystems, Carlsbad, CA, USA). The *AR* CAG repeat was genotyped using fragment analysis. A DNA fragment containing the CAG repeat (at physical position 66,763,874−66,944,119 in chromosome X) in the *AR* gene was amplified using the polymerase chain reaction (PCR) with the forward and reverse primers 5′-ACC GAG GAG CTT TCC AGA AT-3′ and 5′-CTG GGA CGC AAC CTC TCT C-3′respectively. The primers were purchased from Oligomer (Helsinki, Finland) and the forward primer was labeled with the fluorescent 6-FAM dye. The sizes of the PCR fragments were determined using the ABI3130xl Genetic Analyzer (Applied Biosystems), and the GeneScan-500 LIZ Size Standard (Applied Biosystems) was used as a size standard. The fragment lengths were analyzed using the GeneMapper software, v. 3.7 (Applied Biosystems). PCR resulted in fragments with sizes ranging from 300 to 393 bp. To determine the exact number of CAG repeats, a part of the samples were sequenced. Sequencing revealed that fragment analysis had resulted in a 12-bp downwards shift in the actual size of the PCR products. To correct for this bias, the fragment sizes were adjusted for the shift. The shortest and longest PCR fragments (adjusted sizes of 312 and 405 bp) corresponded to 11 and 42 CAG repeats, respectively.

### Statistical Analysis in the Case-Control Study

#### Quality control

Before association analysis, the case-control data was checked for Mendelian inconsistencies using PedCheck [33]. Any inconsistent genotypes were recoded as missing.

#### Analysis of SNP frequencies

SNP allele distributions were analyzed with logistic regression (additive model) with sex as a covariate using PLINK, v. 1.07 [31]. Genotypes of female individuals were also analyzed under the dominant and recessive models. Case-control comparisons of haplotypes were performed using Haploview v. 4.2 [49].

#### Analysis of *AR* CAG*_n_* distribution

Differences in *AR* CAG*_n_* distributions were analyzed using the nonparametric Mann-Whitney *U* test using Predictive Analytics Software (PASW) Statistics, v. 17.0.3 (IBM, SPSS, Inc.). To equalize the contributions of male and female alleles, the tests were performed with the hemizygous males treated as homozygotes. The CAG*_n_* distributions were divided into quintiles and further analyzed using the Pearson *X^2^* test with PASW Statistics. The CAG*_n_* analyses were replicated using biallelic means for the females. Family-based association tests of CAG*_n_* were performed using Pseudomarker, v. 1.0.6b [50], and FBAT, v. 2.0.2c [51], under the dominant model. Pseudomarker was used to test for linkage in the presence of LD (linkage given LD test) in mother-father-offspring trios or mother-offspring pairs; this test is analogous to the model-free transmission disequilibrium test. Pseudomarker can handle missing parental data and different data structures such as mixtures of singletons and families jointly; therefore both the mother-father-affected offspring trios and mother-control offspring pairs are informative in this analysis. FBAT was further used to determine significances in preferential transmission of individual CAG*_n_* alleles in mother-father-affected offspring trios.

#### Significance threshold and power consideration

Using the Bonferroni correction to account for testing of ten polymorphisms (nine SNPs and the *AR* CAG*_n_*), a *p* value ≤0.005 was considered significant in case-control comparisons. Power consideration for the initial case-control study (considering multiple testing of ten polymorphisms, alpha  = 0.005; prevalence of 0.05; MAFs ranging from 0.15 down to 0.05; allelic test under additive model): the sample size was estimated to provide 80% power to detect relative risks of 1.92−2.55 and 2.84−4.10 for the heterozygous and homozygous genotypes, respectively, both in the offspring and mothers.

### Genome Coordinates

Chromosomal positions refer to NCBI Build 37.2 of the human genome.

### Web Resources

1000 Genomes Project: http://www.1000genomes.org.

Genetic Power Calculator: http://pngu.mgh.harvard.edu/~purcell/gpc.

International HapMap project: www.hapmap.org.

## Results

Some of these results were described in a preliminary form in the Ph.D. thesis of the first author [52].

### Linkage Analysis of X chromosomal Markers in SPTB

Seven northern Finnish families with recurrent SPTB were selected for linkage analysis using stringent phenotypic criteria, with deliveries involving known risk factors of SPTB excluded. Because family studies suggest that maternal factors may play an important role in the inheritance of SPTB [8], [12], [13], [16], only families with apparent maternal inheritance were included. Genealogical surveys ensured that the selected families were not consanguineous.

**Figure 2:**
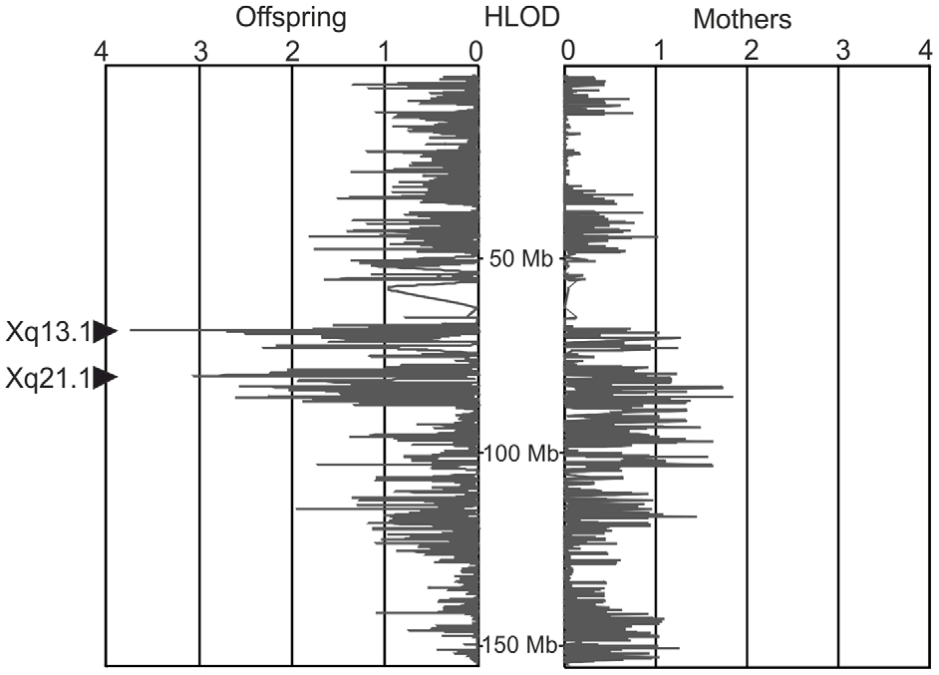
HLOD scores in parametric linkage analysis of X chromosomal markers in spontaneous preterm birth. HLODs for the affected offspring phenotype are shown on the left and those for the affected mother phenotype on the right. The position of the marker yielding a significant linkage signal (SNP rs6525299 on Xq13.1, HLOD of 3.72) for the affected offspring phenotype is shown. The position of the markers yielding the second highest HLODs (SNPs rs11266953 and rs7056400 on Xq21.1, HLODs of 3.05) is also indicated. These three SNPs are intergenic.

Parametric two-point linkage analysis of SPTB was performed using a high-density panel of X chromosomal markers. HLOD scores are depicted in Figure 2. For the affected offspring phenotype, a significant linkage signal was obtained for SNP rs6525299 (θ = 0.00, α = 1.00) on chromosome locus Xq13.1 (HLOD  = 3.72). HLOD scores for the three markers that yielded the highest signals in linkage analysis are shown separately for each of the families in Table 2. Two families (126 and 253) displayed HLOD scores of 0.00 for these markers, indicating that there was no evidence of linkage in these families. For the affected mother phenotype, no significant linkage signals were detected (Figure 2); HLOD_max_ of 1.83 was detected for SNP rs4240068 on Xq21.2.

**Table 2:** SNPs with highest HLOD scores in parametric linkage analysis of spontaneous preterm birth with the affected fetus phenotype.

	rs6525299	rs11266593	rs7056400
**Locus**	Xq13.1	Xq21.1	Xq21.1
**Physical position**	68,623,050	80,124,948	80,173,940
**Overall HLOD**	3.72^a^	3.05^a^	3.05^a^
**HLOD for each family:**			
Family 24	0.42	0.01	0.01
Family 70	1.38	1.72	1.72
Family 126	0.00	0.00	0.00
Family 150	0.69	0.69	0.69
Family 185	0.80	0.20	0.20
Family 210	0.42	0.42	0.42
Family 253	0.00	0.00	0.00

a θ = 0.00, α = 1.00.

### Case-Control Association Analysis of *AR* and *IL2RG* Polymorphisms in the Initial SPTB Study Population Originating from Northern Finland

The genes investigated as candidates for SPTB were selected based on the location of the marker giving the significant signal in linkage analysis. Based on their function, two genes located near the marker yielding the maximum HLOD in linkage analysis (rs6525299) emerged as interesting candidates for SPTB: those encoding androgen receptor (*AR*, MIM *313700, located on Xq12) and interleukin-2 receptor gamma subunit (*IL2RG*, MIM *308380, located on Xq13.1). These genes are located at a distance of approximately 2 Mb from SNP rs6525299.

Case-control association analysis of the selected genes in SPTB was performed in a population consisting of mothers with preterm and term deliveries and their preterm and term-born offspring, respectively. Tagging SNPs representative of common variation within each gene were selected using the HapMap CEU population. In addition, other polymorphisms with potential biological significance were included. For the offspring, the SNP analyses were first performed in mixed-sex samples with sex as a covariate. *IL2RG* SNP rs7880291 was not polymorphic in the study population. Results of logistic regression analysis performed for the *AR* and *IL2RG* SNPs are shown in Table 3; genotype distributions of these polymorphisms did not differ between SPTB and term mothers or offspring. Analysis of female individuals under the dominant and recessive models did not reveal any significant associations (data not shown). Five *AR* SNPs (rs6152, rs5918757, rs5919393, rs5918762, and rs12014709) and two *IL2RG* SNPs (rs4612544 and rs28743771) resided in haplotype blocks. Altogether three 5-SNP *AR* haplotypes and two 2-SNP *IL2RG* haplotypes with a frequency >0.01 were predicted (Table 4). Analysis of these haplotypes did not reveal any significant differences in haplotype frequencies between SPTB offspring or mothers and their controls (Table 4).

**Table 3:** Logistic regression analysis of *AR* and *IL2RG* polymorphisms in SPTB in the initial case-control population.

			Minor allele frequency		Logistic regression (additive model)	
	Gene and polymorphism	Position	SPTB	Term	*p*	OR (95% CI)
**Mothers**			*n* = 272	*n* = 201		
	*AR*, rs6152	exon 1, Glu213Glu	0.146	0.150	0.88	0.97 (0.67–1.42)
	*AR*, rs5918757	intron 1	0.139	0.154	0.55	0.89 (0.60–1.31)
	*AR*, rs5919393	intron 1	0.148	0.154	0.80	0.95 (0.66–1.38)
	*AR*, rs5918762	intron 3	0.139	0.145	0.80	0.95 (0.64–1.41)
	*AR*, rs12014709	intron 5	0.047	0.067	0.22	0.70 (0.39–1.23)
	*AR*, rs5031002	intron 6	0.006	0.025	0.03^a^	0.23 (0.06–0.86)
	*IL2RG*, rs4612544	3′	0.090	0.122	0.11	0.69 (0.45–1.09)
	*IL2RG,* rs7880291	intron 1	0	0	-	-
	*IL2RG*, rs28743771	intron 1	0.095	0.124	0.17	0.73 (0.47–1.15)
**Offspring**			*n* = 269	*n* = 199		
	*AR*, rs6152	exon 1, Glu213Glu	0.126	0.126	1.00	1.00 (0.63–1.60)
	*AR*, rs5918757	intron 1	0.118	0.122	0.87	0.96 (0.58–1.58)
	*AR*, rs5919393	intron 1	0.128	0.126	0.94	1.02 (0.65–1.61)
	*AR*, rs5918762	intron 3	0.106	0.113	0.79	0.93 (0.56–1.56)
	*AR*, rs12014709	intron 5	0.053	0.042	0.55	1.23 (0.62–2.44)
	*AR*, rs5031002	intron 6	0.006	0.020	0.15	0.29 (0.05–1.53)
	*IL2RG*, rs4612544	3′	0.102	0.102	0.99	1.00 (0.60–1.67)
	*IL2RG,* rs7880291	intron 1	0	0	-	-
	*IL2RG*, rs28743771	intron 1	0.103	0.108	0.85	0.95 (0.57–1.59)

a Not significant at the Bonferroni-corrected significance level (*p* ≤ 0.005 considered significant).

**Table 4:** Association analysis of *AR* and *IL2RG* haplotypes in SPTB in the initial case-control population.

			Frequency		
	Gene	Haplotype	SPTB	Term	*p*
**Mothers**			*n* = 272	*n* = 201	
	*AR* a	GAACT	0.851	0.847	0.85
		AGGTT	0.098	0.087	0.57
		AGGTG	0.049	0.064	0.33
	*IL2RG* b	AA	0.907	0.876	0.13
		GG	0.091	0.122	0.14
**Offspring**			*n* = 269	*n* = 199	
	*AR* a	GAACT	0.880	0.874	0.83
		AGGTT	0.067	0.082	0.47
		AGGTG	0.047	0.043	0.83
	*IL2RG* b	AA	0.899	0.891	0.73
		GG	0.098	0.102	0.85

a SNPs in the haplotype: rs6152, rs5918757, rs5919393, rs5918762 and rs12014709.

b SNPs in the haplotype: rs4612544 and rs28743771.

The distributions of *AR* CAG repeat numbers in each group studied are shown in Figure S1. The observed distributions are consistent with those previously reported for individuals of European descent [53]. Nearly all of the CAG*_n_* alleles in our population were in the range of 13–30, which falls in the normal range of repeat lengths (10–36). Very long alleles (38 or 42 repeats) were detected in four individuals (two SPTB mothers and their SPTB offspring). The median number of *AR* CAG repeats was 22 in all groups studied, except in female SPTB offspring, who had median of 23. The distribution of CAG repeats differed significantly between individuals born preterm and those born at term (Mann Whitney *U* test, *p* = 0.0006), with carriers of the longest repeats (≥26) being at a more than 2-fold risk (OR  = 2.45, 95% CI 1.46–4.13) of being born preterm compared to the carriers of the shortest repeats (≤19) (Table 5), suggesting that longer alleles may predispose to SPTB. The difference was clearer in females than in males (Table S2). Differences in CAG*_n_* distributions between SPTB and term offspring were also significant when biallelic means were used in the comparisons (data not shown). There was no significant difference in the *AR* CAG*_n_* distribution between SPTB mothers and mothers with term deliveries (Mann-Whitney *U t*est, *p* = 0.13; mean ± standard deviation, SD, 22.41±3.02 vs. 22.06±2.81, respectively). The fathers of the preterm offspring had a CAG*_n_* mean of 22.15±2.75.

**Table 5:** *AR* CAG repeat distributions in individuals born spontaneously preterm and in those born at term.

	*n*	Mean ± SD^a^	Mann-Whitney *U* test, *p*	Frequency of repeats in quintiles^b^	*X^2^* test for quintiles, *p*	OR (95% CI)^c^
**Oulu:**						
SPTB	252	22.59±3.11	0.0006	0.109/0.282/0.260/0.208/0.141	0.009	2.45 (1.46–4.13)
Term	189	21.87±2.64		0.201/0.267/0.275/0.151/0.106		
**Helsinki:**						
SPTB	111	22.45±3.35	0.67	0.122/0.338/0.216/0.180/0.144	0.047	2.25 (1.14–4.43)
Term	197	22.09±2.54		0.145/0.305/0.269/0.206/0.076		
**Combined Oulu-Helsinki:**						
SPTB	363	22.55±3.18	0.0027	0.113/0.299/0.247/0.200/0.142	0.0006	2.39 (1.58–3.60)
Term	386	21.98±2.59		0.172/0.286/0.272/0.179/0.091		

a Mean ± standard deviation of CAG*_n_* repeat length.

b 1^st^ quintile 10–19 repeats, 2^nd^ quintile 20–21 repeats, 3^rd^ quintile 22–23 repeats, 4^th^ quintile 24–25 repeats, 5^th^ quintile 26–42 repeats; frequencies shown separately for male and female individuals in Table S2.

c ORs for the highest quintile relative to the lowest quintile.

To evaluate whether the predisposing *AR* CAG*_n_* alleles had been inherited from the mother or the father, distributions of maternally and paternally inherited alleles were compared between SPTB and term offspring. This revealed that distributions of both maternally and paternally inherited CAG*_n_* alleles differed between cases and controls; the difference was more significant for the maternal alleles (Mann Whitney *U* test, *p* = 0.0045, mean ± SD, 22.59±3.19 *vs.* 21.74±2.63, respectively, for maternal alleles; Mann Whitney *U* test, *p* = 0.040, 22.77±2.82 *vs.* 21.84±2.72, respectively, for paternal alleles). When the repeat lengths were divided into quintiles, under- and overrepresentation of the lowest and highest groups, respectively, could be seen in both maternally and paternally inherited alleles (data not shown).

Family-based tests were performed to further investigate whether specific CAG*_n_* alleles had been preferentially transmitted to affected individuals, i.e. offspring born preterm. The linkage given LD test showed evidence for disequilibrium in the transmission of *AR* CAG*_n_* alleles (*p* = 0.004), indicating that this polymorphism is linked to the affected offspring phenotype in the families of the case-control population. FBAT analysis further suggested that the allele with 19 repeats had been undertransmitted and the allele with 24 repeats overtransmitted to preterm-born offspring (Table 6), again suggesting that shorter alleles may be protective and longer alleles predisposing to the affected offspring phenotype.

**Table 6:** FBAT analysis of the *AR* CAG*_n_* polymorphism under the dominant model in mother-father-preterm offspring trios of the initial SPTB case population.

CAG*_n_* allele (number of repeats)^a^	Number of families	*Z* b	*p*
18	31	−0.180	0.857
19	20	−2.236	0.025
20	42	1.234	0.217
21	56	−1.336	0.181
22	42	1.234	0.217
23	41	−0.469	0.639
24	28	1.890	0.059
25	46	0.000	1.000
26	25	−0.200	0.841
27	11	−0.905	0.366

a Analysis was not performed for alleles with less than 10 transmissions.

b Positive and negative values of the FBAT test statistic *Z* are indicative of over- and undertransmission, respectively.

Allele or haplotype frequencies of any of the studied polymorphisms, including *AR* CAG*_n_*, did not differ between SPTB mothers or offspring with or without PPROM, according to gender, or between individuals born very preterm (gestational age <32 wk) and moderately preterm (gestational age ≥32 wk) (data not shown). The frequencies and *AR* CAG_n_ distributions were similar in offspring from families with multiple and single SPTBs (data not shown).

### Case-Control Association Analysis of *AR* and *IL2RG* Polymorphisms in the Replication SPTB Study Population Originating from Southern Finland

Because association analyses performed in the initial case-control study population revealed an association of a fetal polymorphism with SPTB, the case-control analyses were repeated in a study population consisting of preterm and term offspring originating from the southern Finland, where the population is known to be genetically more diverse compared to northern Finland [54]. Similarly to the initial population, none of the analyzed *AR* and *IL2RG* SNPs associated with SPTB in the replication population (data not shown). The range of the *AR* CAG*_n_* repeats was 11–40. The allele with 40 repeats was detected in a single individual born preterm while rest of the alleles were ≤32 repeats in length. Similarly to the population of northern Finland, the longer alleles tended to be more frequent in SPTB than in term offspring but the difference in the distribution was not significant (Mann Whitney *U* test, *p* = 0.67; Table 5). However, similarly to the northern Finnish population, carriers of the longest CAG repeats (≥26) were at a more than 2-fold risk (OR 2.25, 95% CI 1.14–4.43) of being born preterm compared to the carriers of the shortest repeats (≤19) (Table 5), further suggesting that the long fetal *AR* CAG*_n_* alleles may be involved in giving predisposition to SPTB. Finally, when the populations of northern and southern Finland were combined, the difference in CAG*_n_* distribution between SPTB and term offspring was significant (Mann Whitney *U* test, p = 0.0027; Table 5).

## Discussion

We recently reported the first linkage analysis of SPTB, which was performed for autosomal markers in seven families with recurrent SPTB [27]. In the present study, linkage analysis of X chromosomal markers in these same families revealed a significant linkage signal on chromosome locus Xq13.1 (HLOD of 3.72 for SNP rs6525299) when the phenotype was being born preterm (affected offspring phenotype). Two genes near this locus, *AR* and *IL2RG*, were investigated in subsequent case-control association analysis of mothers with preterm and term deliveries and their offspring. Finally, our results indicated fetal *AR* as a novel gene potentially involved in genetic predisposition to SPTB.

The length of the *AR* exon-1 CAG repeat was associated with SPTB in the offpring, with longer repeat lengths overrepresented in individuals born preterm compared to those born at term (Table 5). AR is a nuclear receptor for the androgens testosterone or dihydrosterone. It functions as a transcription factor regulating expression of its target genes involved in the development of several tissue, in maintenance of male sexual characteristics, and in female reproductive physiology [40]. AR also has non-genomic actions through interactions with signal transduction proteins in the cytoplasm [55], [56]. The *AR* CAG repeat encodes a polyglutamine tract present in the transactivation domain of AR [46]. This domain is involved in ligand-induced transcriptional activity. Longer polyglutamine chains have been shown to hinder the transactivation activity of AR *in vitro*, while short polyglutamine chains lead to enhanced AR activation resulting in hyperandrogenism [46], [57]–[59]. Thus, it is evident that the CAG repeat length polymorphism has biologically significant consequences for AR function.

AR has important roles during pregnancy and may be involved in some pregnancy-related conditions. AR is widely expressed in reproductive tissues of both males and females [60]. In developing first-trimester human embryos, expression of AR has been detected in several tissues, including several extragenital sites, such as thymus, bronchial epithelium of the lung, spinal cord, and cardiac valves [61]. AR levels in the fetal lung change significantly with gestational age during mid- and late gestation [62]. Longer *AR* CAG repeats (>19) have been shown to be overrepresented in women with recurrent spontaneous abortions [48], and increased *AR* expression has been detected in placentas of preeclamptic women [63], suggesting that AR plays a role in these pregnancy-related complications. Furthermore, successful induction of labor by local prostaglandin treatment leads to decreased AR and progesterone receptor levels in human uterine cervix [64]. This suggests that besides the decrease in progesterone activity, androgen withdrawal may be involved in the onset of parturition. However, complete androgen insensitivity syndrome of the fetus does not seem to lead to preterm birth [65]. Further studies on the roles of *AR* exon-1 CAG repeat and the genes regulated by *AR* could lead to further understanding of the function of fetal AR in regulation of the onset of labor.

In our previous linkage study performed in the same families as the present analysis, we found a significant linkage signal within the gene encoding *IGF1R* and identified a 6-SNP haplotype within this gene as a fetal susceptibility factor for SPTB [27]. Interestingly, recent studies have identified *IGF1R* as a downstream target for AR [66], [67]. Furthermore, androgens have been shown to be involved in activation of *IGF1R* through non-genomic pathways involving AR [67]–[69]. In addition, hypermethylation of the *AR* promoter may be involved in the progression of prostate cancer and downregulate expression of *IGF1R* [67]. Because these two genes were identified as fetal susceptibility factors for SPTB in our previous and current studies, it is tempting to postulate that interactions of these genes may affect the onset of spontaneous preterm labor. However, further studies are needed to test this hypothesis.

In the present study, we did not find significant linkage in the mothers, i.e. when the phenotype was giving preterm deliveries. This may be due to the small sample numbers in this analysis (*n_affected_*  = 21) leading to low power to detect linkage. Previous genetic modeling studies have estimated that both maternal and fetal genetic effects contribute to the timing of parturition [15]–[17]. This is consistent with our finding of a fetal gene associated with SPTB. However, several large population-based studies suggest a significant contribution of maternal genes to preterm delivery with little effect of fetal genes [8], [12], [13]. In a Dutch twin study, there was no detectable evidence for paternal heritability of parturition timing [70]. These studies suggest that paternal genes acting in the fetus have little effect on the risk of preterm birth compared to maternal genes acting either in the pregnant mother or in the fetus. It remains to be investigated whether both maternal and paternal alleles of the fetus could contribute to the SPTB phenotype.

The strengths of our study include 1) the use of the family-based linkage analysis for localization of the associating genomic region, 2) the use of the northern Finnish population, which is known to be genetically relatively homogeneous [54], 3) further analysis in the genetically more diverse southern Finnish population, 4) analysis of both the fetal and maternal contributions, and 5) stringent selection criteria for the SPTB phenotype with deliveries involving major risk factors for preterm birth excluded. In addition, our initial control population consisted of mothers with exclusively at least three term deliveries, and their offspring. These individuals therefore represented the so-called “super controls” for the SPTB phenotype. To avoid false positives in the case-control study, the significances were considered on the stringent Bonferroni-corrected level.

The limitations of our study include the relatively small number of families in linkage analysis and the relatively small sample sizes of both the initial case-control study population and particularly that of the replication study population. Although association between the fetal *AR* CAG repeat and SPTB was not directly replicated in the southern Finnish population, the quintile comparison suggested that, similarly to the northern Finnish population, the longest alleles may be overrepresented in individuals born preterm (Table 5). This analysis was apparently limited by the small sample sizes in each of the quintile subgroups in the southern Finnish population. However, because the differences in the *AR* CAG distribution were significant when the northern and southern Finnish populations were combined, we feel that results of the southern Finnish population strengthened the evidence for the role of fetal *AR* CAG repeat in genetic predisposition to SPTB. Our study was also limited in that we only analyzed two genes located near the linkage signal. Thus, we cannot exclude the possibility that other genes located in the linked region could play a role in SPTB. We also cannot exclude a possible role for genes located near the suggestive linkage signals on chromosome locus Xq21.1 (Figure 2) in SPTB. In the current setting, we cannot evaluate whether the linkage signal actually was due to linkage to *AR* CAG*_n_* polymorphism. Short tandem repeat polymorphisms (STRPs), such as *AR* CAG*_n_*, are known to display significant LD with SNPs even on long distances (up to 2 cM), and STRP-SNP LD is greater on the X chromosome compared to autosomes [44]. Therefore, it is possible that *AR* CAG*_n_* polymorphism indeed is linked to SPTB. This conclusion is further supported by the significant result of the family-based linkage given LD test performed in the northern Finnish case-control families in the present study. Further studies are needed to evaluate the association between the fetal *AR* CAG repeat polymorphism and SPTB; these should include replication in other, more outbred populations and larger sample sizes.

In conclusion, we found the highest linkage signal (HLOD  = 3.72) on chromosome Xq13.1 when the phenotype was being born preterm. Two genes located near this locus, encoding the androgen receptor (AR) and the interleukin-2 receptor gamma subunit (IL2RG), were considered functional candidates for SPTB and were thus analyzed in a subsequent case-control association analysis of mothers and their offspring originating from northern Finland, and in an independent case-control population of offspring from southern Finland. Finally, we demonstrated that a CAG repeat polymorphism present in exon 1 of the *AR* gene associates with SPTB in the offspring. Therefore, our results implicate *AR* as a potential fetal susceptibility gene for SPTB. The role of AR in parturition timing remains to be investigated in subsequent studies.

## Supporting Information

Figure S1 ***AR*** **CAG repeat distributions in the initial case-control population.**
(PDF)

Table S1 **Gestational ages and years of birth for the preterm-born members of the seven families with recurrent SPTB analyzed in the linkage analysis of X chromosomal markers.**
(PDF)

Table S2 ***AR*** **CAG repeat distributions in male and female SPTB and term offspring.**
(PDF)
