# Supplementary figures and images for: A Potential Novel Spontaneous Preterm Birth Gene, AR, Identified by Linkage and Association Analysis of X Chromosomal Markers
Source: PLoS One. 2012 Dec 5;7(12):e51378. doi: 10.1371/journal.pone.0051378 (PMC3515491; doi:10.1371/journal.pone.0051378)

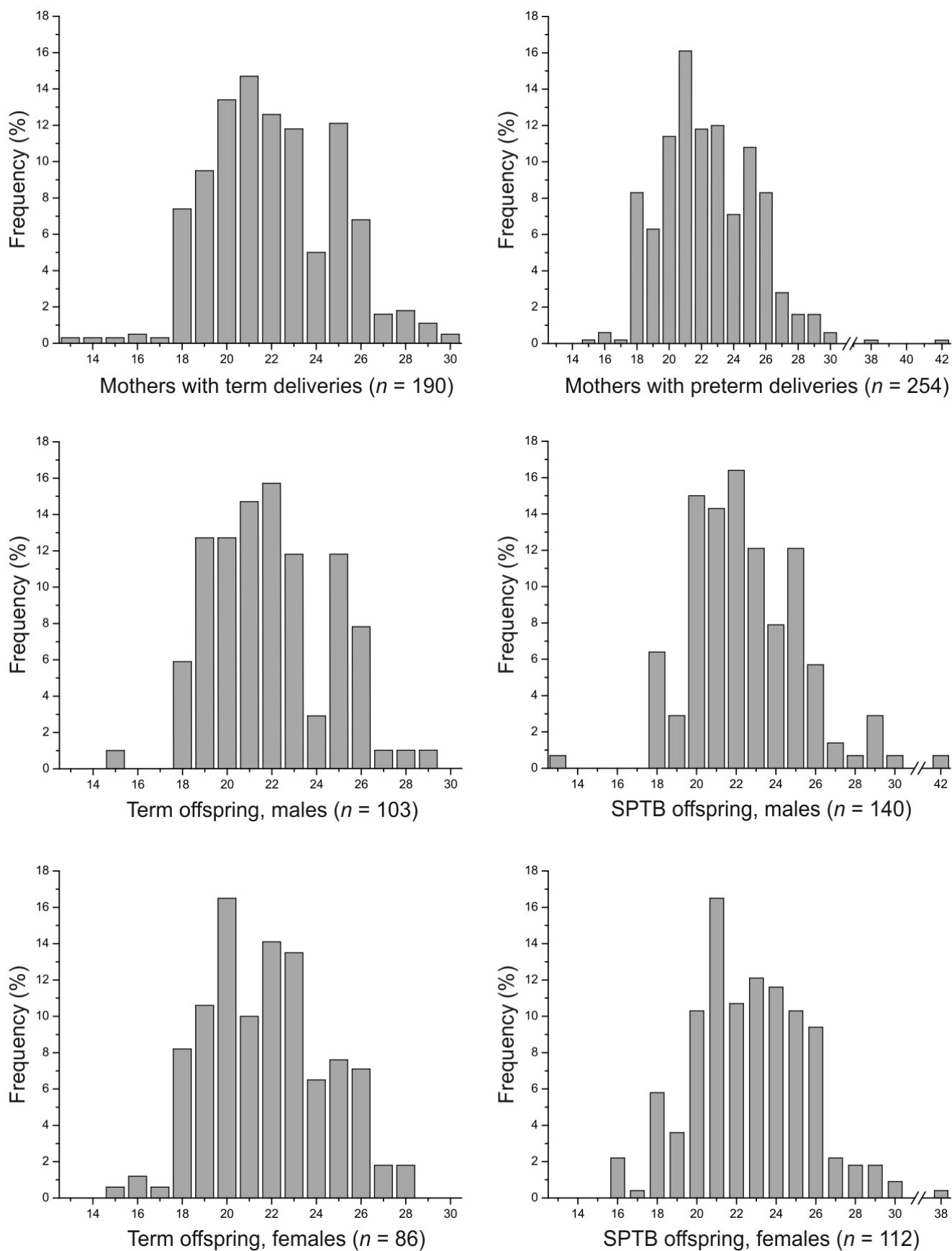

**Figure S1. AR CAG repeat distributions in the initial case-control population.**

Supplement: Figure S1 — AR CAG repeat distributions in the initial case-control population. (PDF) [file pone.0051378.s001.pdf]
