# Supplementary material for: A Potential Novel Spontaneous Preterm Birth Gene, AR, Identified by Linkage and Association Analysis of X Chromosomal Markers
Source: PLoS One. 2012 Dec 5;7(12):e51378. doi: 10.1371/journal.pone.0051378 (PMC3515491; doi:10.1371/journal.pone.0051378)
Supplement: Table S1 — Gestational ages and years of birth for the preterm-born members of the seven families with recurrent SPTB analyzed in the linkage analysis of X chromosomal markers. (PDF) [file pone.0051378.s002.pdf]

Table S1. Gestational ages and years of birth for the preterm-born members of the seven families with recurrent SPTB analyzed in the linkage analysis of X chromosomal markers.

Family	Individual ID[a]	Gestational age (weeks+days)[b]	Year of birth
24	3	35+0	1948
24	4	35+0	1956
24	8	32+0	1970
24	10	35+5	1985
70	2	35+0	1948
70	4	32+0	1952
70	5	35+0	1953
70	8	32+3	1984
70	10	34+4	1989
70	11	27+6	2006
126	6	33+0	1975
126	7	35+0	1976
126	8	34+6	1993
150	5	35+6	1941
150	9	34+0	1955
150	11	34+0	1957
150	12	35+6	1958
150	13	35+6	1959
150	14	34+1	1973
150	15	34+0	1974
150	16	33+1	1978
150	18	32+0	1968
150	19	34+0	1969
150	20	34+3	1974
150	23	32+3	1982
150	24	34+4	1983
150	25	32+0	1985
150	27	31+5	1995
150	28	35+0	1996
150	29	35+0	1999
150	30	34+0	2006
185	7	35+6	2003
185	8	33+2	2003
185	10	32+2	1991
210	2	34+0	1958
210	7	35+6	1988
210	8	34+4	1997
253	6	35+0	1980
253	7	35+6	1985
253	8	32+1	1986
253	9	33+3	1991

[a] The preterm-born individuals are coded as indicated in Figure 1

[b] Gestational ages of individuals born before 1973 were estimated based on family interviews
