# Supplementary material for: A Potential Novel Spontaneous Preterm Birth Gene, AR, Identified by Linkage and Association Analysis of X Chromosomal Markers
Source: PLoS One. 2012 Dec 5;7(12):e51378. doi: 10.1371/journal.pone.0051378 (PMC3515491; doi:10.1371/journal.pone.0051378)
Supplement: Table S2 — AR CAG repeat distributions in male and female SPTB and term offspring. (PDF) [file pone.0051378.s003.pdf]

Table S2. *AR* CAG repeat distributions in male and female SPTB and term offspring.

Study group	*n*	Mean ± SD[a]	Mann-Whitney *U* test, *p*	Frequency of repeats in quintiles[a]	X^2 test for quintiles, *p*	OR (95% CI)[b]
Oulu offspring, males						
SPTB	140	22.54 ± 3.19	0.170	0.100 / 0.293 / 0.286 / 0.200 / 0.121	0.324	2.21
Term	103	21.95 ± 2.83		0.194 / 0.272 / 0.272 / 0.155 / 0.107		(0.80–6.13)
Oulu offspring, females						
SPTB	112	22.66 ± 3.03	0.003	0.121 / 0.268 / 0.228 / 0.219 / 0.165	0.024	2.74
Term	86	21.77 ± 2.67		0.209 / 0.262 / 0.279 / 0.145 / 0.105		(1.29–5.82)
Helsinki offspring, males						
SPTB	56	22.61 ± 3.68	0.387	0.107 / 0.339 / 0.268 / 0.161 / 0.125	0.553	3.50
Term	94	21.78 ± 2.42		0.160 / 0.330 / 0.287 / 0.170 / 0.053		(0.79–15.50)
Helsinki offspring, females						
SPTB	55	22.30 ± 3.01	0.629	0.136 / 0.336 / 0.164 / 0.200 / 0.164	0.172	1.62
Term	103	22.38 ± 2.62		0.131 / 0.282 / 0.252 / 0.238 / 0.097		(0.66–3.97)
Combined Oulu-Helsinki offspring, males						
SPTB	196	22.56 ± 3.33	0.079	0.102 / 0.306 / 0.281 / 0.189 / 0.122	0.195	2.63
Term	197	21.87 ± 2.53		0.178 / 0.299 / 0.279 / 0.162 / 0.081		(1.14–6.07)
Combined Oulu-Helsinki offspring, females						
SPTB	167	22.54 ± 3.02	0.068	0.126 / 0.290 / 0.207 / 0.213 / 0.165	0.032	2.17
Term	189	22.11 ± 2.66		0.167 / 0.272 / 0.265 / 0.196 / 0.101		(1.23–3.83)

[a] Mean ± standard deviation of CAG_n repeat length
[b] 1st quintile 10-19 repeats, 2nd quintile 20-21 repeats, 3rd quintile 22-23 repeats, 4th quintile 24-25 repeats, 5th quintile 26-42 repeats
[c] ORs for the highest quintile relative to the lowest quintile
